# A Robust Liquid Chromatographic Method for Confirmation of Drug Stability of Azithromycin in Bulk Samples, Tablets and Suspensions

**DOI:** 10.3390/pharmaceutics9010011

**Published:** 2017-02-24

**Authors:** Alex O. Okaru, Kennedy O. Abuga, Franco N. Kamau, Stanley N. Ndwigah, Dirk W. Lachenmeier

**Affiliations:** 1 Department of Pharmaceutical Chemistry, University of Nairobi, Off Ngong Road, Nairobi, P.O. Box 19676-00202 Nairobi, Kenya; koabuga@uonbi.ac.ke (K.O.A.); franco.kamau@uonbi.ac.ke (F.N.K.); snndwigah@uonbi.ac.ke (S.N.N.); 2 Chemisches und Veterinäruntersuchungsamt (CVUA) Karlsruhe, Weissenburger Straße 3, 76187 Karlsruhe, Germany; lachenmeier@web.de

**Keywords:** azithromycin, HPLC, robust, isocratic, drug stability, degradation products

## Abstract

A simple, isocratic and robust RP-HPLC method for the analysis of azithromycin was developed, validated and applied for the analysis of bulk samples, tablets and suspensions. The optimum chromatographic conditions for separation were established as a mobile phase comprised of acetonitrile-0.1 M KH_2_PO_4_ pH 6.5–0.1 M tetrabutyl ammonium hydroxide pH 6.5-water (25:15:1:59 *v*/*v*/*v*/*v*) delivered at a flow rate of 1.0 mL/min. The stationary phase consisted of reverse-phase XTerra^®^ (250 mm × 4.6 mm i.d., 5 µm particle size) maintained at a temperature of 43 °C with a UV detection at 215 nm. The method was found to be linear in the range 50%–150% (*r*^2^ = 0.997). The limits of detection and quantification were found to be 0.02% (20 µg) and 0.078% (78 µg), respectively, with a 100.7% recovery of azithromycin. Degradation products of azithromycin in acidic and oxidative environments at 37 °C were resolved from the active pharmaceutical ingredient and thus the method is fit for the purpose of drug stability confirmation.

## 1. Introduction

**Figure 1:**
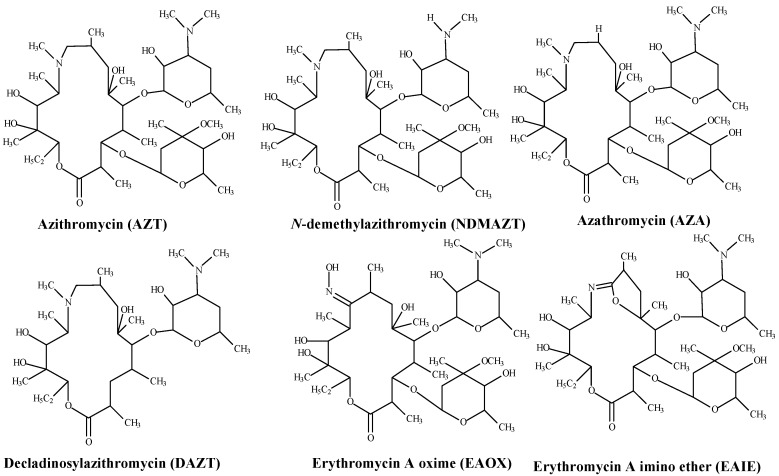
Chemical structures of azithromycin and some related substances.

Azithromycin is a semi-synthetic macrolide antibiotic used clinically for a wide range of bacterial infections [1,2]. Azithromycin is listed in the World Health Organization and the Ministry of Health, Kenya essential medicines lists [3,4] where it is indicated for the management of atypical infections. Suitable analytical methods are required to monitor the quality of azithromycin (AZT) products during manufacture, batch release and post-market surveillance. The analytical method ought to be selective for AZT in the presence of related substances, namely the synthetic intermediates azathromycin (AZA), erythromycin A oxime (EAOX) and erythromycin A imino ether (EAIE) and the degradation products *N*-demethylazithromycin (NDMAZT) and decladinosylazithromycin (DAZT) (Figure 1).

There are several published methods for the analysis of azithromycin such as biological methods [5], electroanalytical methods [6], spectrophotometric methods [7], capillary electrophoresis [8], thin layer chromatography [9] and liquid chromatography [10,11,12,13]. An excellent review of the analytical methods for azithromycin is reported by Sharma and Mullangi [14]. Liquid chromatography is the method of choice for the analysis of azithromycin in bulk samples and formulations since it is able to determine azithromycin in the presence of related substances. The current liquid chromatographic methods for the analysis of azithromycin utilize relatively expensive columns, unstable detectors and high pH and/or temperature, limiting the use of conventional silica-based columns [12,13,15]. The United States Pharmacopoeia [13] employs a mobile phase of pH 11 with electrochemical detection while the British Pharmacopoeia [12] utilizes a polymer column with a mobile phase of pH 8. The electrochemical detector is not commonly available and produces unstable responses compared to the UV detector since the electrodes have direct contact with the column effluent and may react with the sample matrix while polymer columns are relatively more expensive compared to silica-based columns. This calls for use of pH- and temperature-stable strategies to improve on column longevity, stability and peak parameters. Such a method can be used for the routine quality control of azithromycin bulk samples and formulations as well as the market surveillance of samples. A reverse-phase high-performance liquid chromatographic method for the estimation of azithromycin suspension described by Sachin et al. [16] did not exhibit the required specificity regarding the related substances NDMAZT, AZA, DAZT, EAOX and EAIE, while the gradient method proposed by Miguel and Barbas [17] suffers from baseline instability and uses a slightly high temperature of 50 °C. This paper describes the development, validation and application of a selective, isocratic and robust liquid chromatographic method for the analysis of azithromycin in bulk samples and oral dosage forms. 

## 2. Materials and Methods 

### 2.1. Chemicals and Reagents

Analytical grade KH_2_PO_4_, K_2_HPO_4_ and H_3_PO_4_ were from Loba Chemie Pvt. Ltd. (Mumbai, India) while triethylamine and triethanolamine were from BDH Laboratory Supplies (Poole, UK). Tert-butylammonium hydroxide was from Sigma-Aldrich (Steinheim, Germany). HPLC grade acetonitrile (Scharlau, Barcelona, Spain) was used as organic modifier. Purified water was prepared using distillation by GFL distillation machine type 2001/4 (Gesellschaft für Labortechnik mbH, Burgwedel, Germany). Azithromycin dihydrate working standard was donated by the Drug Analysis and Research Unit, University of Nairobi, Kenya. The working standards of erythromycin A oxime, erythromycin A imino ether, *N*-demethylazithromycin, decladinosylazithromycin and azathromycin standards were purchased from Pfizer Inc. (Groton, CT, USA). Azithromycin tablets and suspensions were randomly purchased from various pharmacies in the Central Business District of the City of Nairobi. The innovator brand, Zithromax^®^, was from Pfizer Laboratories Ltd. (Nairobi, Kenya). Azithromycin bulk sample was from SkyLight Chemicals (Nairobi, Kenya).

### 2.2. HPLC Instruments and Analytical Conditions

A Shimadzu high performance liquid chromatographic (HPLC) system (Shimadzu Corp., Kyoto, Japan) was used during the study. It was supported by a CBM-20A Prominence communication bus module as the system controller and an LCSolutions software Ver. 1.22, SP1 and equipped with an SPD-M20A Prominence UV/Visible photo array diode detector operating with deuterium and a tungsten lamps as UV and Visible light sources. A LC-20AD Prominence solvent delivery system with a dual-plunger tandem-flow solvent delivery system and a SIL-20AC Prominence autosampler were a part of the HPLC system. The temperature was controlled using a CTO-M20AC column oven with a block heating thermostatic chamber. Mobile phases were degassed using a Power sonic 410 bench-top ultrasonic bath (Daihan Labtech Ltd., Kyonggi-Do, Korea). A Waters XTerra^®^, a hybrid, reverse-phase C18 column (250 mm × 4.6 mm, 5 μm) (Waters Corporation, Milford, MA, USA) was used for separations. The detection wavelength was set at 215 nm while the sample injection volume was at 20 µL and the flow rate at 1 mL/min.

### 2.3. Preparation of Solutions

#### 2.3.1. Working Standard Mixture

The working standard mixture was prepared by dissolving the working standards in acetonitrile-water (50:50 *v*/*v*) to concentrations of azithromycin 5.0 mg, 1.5 mg azathromycin, 0.64 mg demethylazithromycin, 0.75 mg decladinosylazithromycin, 0.304 mg erythromycin A oxime and 0.31 mg erythromycin A imino ether in 1 mL of solution. These concentrations were preferred since they yielded comparable peak heights among the related substances for accurate derivation of chromatographic parameters. The related substances have varying response factors compared to AZT. Thus erythromycin A imino ether was incorporated at 2.5% level while decladinosylazithromycin and azathromycin were at 10% level while maintaining azithromycin at 100% (5 mg/mL). The other related substances were incorporated into the working mixture at levels ranging between 2.5% to 10% for ease of monitoring of chromatographic parameters of all components.

#### 2.3.2. Mobile Phases

Mobile phases were prepared by mixing appropriate volumes 0.1 M KH_2_PO_4_ (stock solution) with water and acetonitrile. The pH of 0.1 M KH_2_PO_4_ had been preadjusted with an equimolar solution of K_2_HPO_4_. Mobile phases were degassed in an ultra-sonic water bath for 30 min before use.

### 2.4. Peak Purity Analysis

Peak purity analysis was performed over a wavelength range of 200–900 nm using the Shimadzu LCSolutions software Ver. 1.22 SP1 (Shimadzu, Kyoto, Japan) [18]. Although peak purity analysis does not completely prove that the peak observed is due to one component, it gives confidence in the method [19].

### 2.5. Method Validation 

#### 2.5.1. Linearity

Linearity was determined on solutions of azithromycin working standard within the 50%–150% range. For this purpose, a 5.0 mg/mL solution was taken as 100%. Solutions were made by dissolving azithromycin in acetonitrile-water (50:50 *v*/*v*) and run in triplicate. The data obtained from the linearity experiments was subjected to linear regression analysis with the concentration of injected AZT standard being plotted against the peak areas obtained.

#### 2.5.2. Sensitivity

The detection limit and limit of quantitation were determined by serially diluting 5 mg/mL AZT solution to yield signal to noise (S/N) ratios of 3 and 10 respectively [20]. 

#### 2.5.3. Accuracy

Accuracy was evaluated by analyzing synthetic mixtures spiked with known quantities of azithromycin. Triplicate determinations at three concentration levels (80%, 100% and 120%) were used. The mean recoveries of the assays were assessed for compliance with the International Conference on Harmonization (ICH) guidelines [20].

#### 2.5.4. Robustness

The Box-Wilson Design (BWD) was used to determine the optimum range of the chromatographic factors within which the method was robust. A three-level (−1, 0 and +1) experimental design was applied out as shown in Table 1. The experiments were randomized with the aid of the Statgraphics Centurion XVI (StatPoint Technologies Inc., Warrento, VA, USA) software. The interactive effects of the three variables of temperature, acetonitrile concentration and buffer pH on the selectivity of the critical peak pair of EAIE and AZT were evaluated as a measure of robustness. Pareto analysis was used to identify the interactive effects that had the greatest impact (*p* < 0.05) on the selectivity of the critical pair of EAIE and AZT.

#### 2.5.5. Precision

The parameters of repeatability and intermediate precision were evaluated as indicators of precision according to ICH guidelines [20]. Repeatability was determined by a six times injection of azithromycin working solution while intermediate precision was evaluated by injecting five replicate solutions of azithromycin working standard over three consecutive days. A coefficient of variation (CV) of <1.5% of the replicate injections was used as an acceptance criteria for method precision [20].

#### 2.5.6. Specificity

The ability of the proposed analytical method to unequivocally assess azithromycin in the presence of the related substances was investigated by determining the resolution between azithromycin and all other components according to ICH guidelines [20].

### 2.6. Assay of Azithromycin in Bulk Samples, Tablets and Suspensions

Twenty tablets were weighed and pulverized using a mortar and pestle and powder equivalent to 250 mg azithromycin was transferred into a 50 mL volumetric flask and dissolved in 25 mL acetonitrile under sonication for 15 min before making up to volume with distilled water. The samples were filtered through a 0.45 µm membrane filter before chromatography.

Suspensions equivalent to 40 mg/mL were weighed into 5 mL volumetric flasks and dissolved in 2.5 mL of acetonitrile under sonication for 15 min before making up to volume with distilled water. The sample was filtered through a 0.45 µm membrane filter before chromatographic analysis. Azithromycin bulk sample was prepared by dissolving 50 mg in 25 mL of acetonitrile under sonication before diluting to 50 mL with distilled water. The test and standard solutions were run under the optimum HPLC conditions and the peak areas obtained were used for the determination of the content of AZT in the samples. 

### 2.7. Forced Degradation and Stability-Indicating Study

Solutions of azithromycin working standard were subjected to stress testing under oxidative and acidic conditions. The stability indicating ability of the method was tested under these two conditions.

#### 2.7.1. Oxidative Degradation of Azithromycin

Oxidation was carried out using 0.0005% *v*/*v* H_2_O_2_ as described by Abuga et al. for clarithromycin [21] and the solutions sampled at defined intervals for HPLC analysis. For this purpose, azithromycin 5 mg/mL solution was incubated in 0.0005% *v*/*v* H_2_O_2_ at 37 °C and the peak areas of AZT were monitored over a period of 7 h. To determine the order of reaction, the data was fitted into zero, first and second order kinetic models whereby the r^2^ value of the linear plots was used as a measure of goodness of fit. 

#### 2.7.2. Degradation of Azithromycin in Acid

Acid degradation was carried out by incubating a 5 mg/mL solution of AZT in 0.1 M H_3_PO_4_ (pH 1.60), 0.05 M H_3_PO_4_ (pH 1.68), 0.025 M H_3_PO_4_ (pH 1.92) and 0.01 M H_3_PO_4_ (pH 2.37), respectively, at 37 °C using the procedure previously described in literature [21]. The peak area of AZT was monitored by HPLC-UV under the optimized conditions. 

## 3. Results and Discussion

### 3.1. Method Development and Optimization

The development of the HPLC method followed a systematic manipulation of the chromatographic factors of pH, temperature, organic modifier concentration and buffer concentration. The process involved the selection of appropriate conditions and their optimization. These conditions included the type of column packing, column dimensions, mobile phase composition and flow rate, oven temperature, sample amount and detection wavelength. A detection wavelength of 215 nm was selected on account of better baseline stability while maintaining acceptable sensitivity. 

The UV spectra obtained for the impurities used in this study did not show appreciable absorption differences. Acetonitrile was selected as the organic modifier because it has a higher eluting power and a lower UV cut-off compared to methanol. A Waters XTerra^®^ hybrid, reverse-phase C18 column (250 mm × 4.6 mm, 5 μm) was chosen for this study since it gave the highest efficiency with regard to the number of theoretical plates compared to Phenomenex Luna^®^ and Phenomenex Gemini^®^ of similar dimensions.

**Figure 2:**
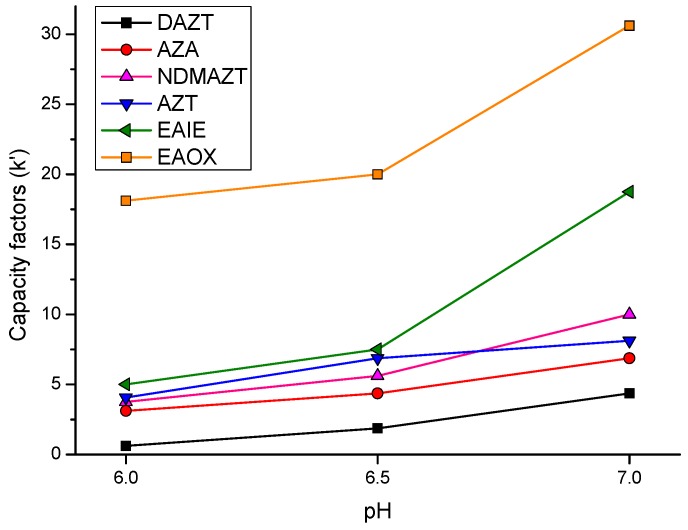
Effect of buffer pH on capacity factors of components of the working mixture.

The effect of adjusting the pH of the phosphate buffer on the separation and symmetry of components was investigated using mobile phases at pH 5.0, 6.0, 6.5 and 7.0. A plot of capacity factor versus pH (Figure 2) revealed that an increase in buffer pH improved the selectivity and also increased the retention times of all the components. Furthermore, an improved peak shape was observed with the increase in pH. However, at pH 7.0 there was co-elution of AZT and EAIE while at pH 6.0 there was poor resolution between AZT and *N*DMAZT. Through several experiments, AZT and EAIE were identified as the critical peak pair (CPP) and it was used as an indicator of the method’s performance.

To improve the separation between AZT and EAIE, the proportion of the organic modifier was reduced to 20% while maintaining pH 6.5. Under these chromatographic conditions, partial resolution was achieved, although the run time increased from 60 to 100 min. To achieve baseline resolution, the incorporation of tetrabutylammonium hydroxide at a concentration of 1% led to a shorter run time (45 min) but with decreased resolution of decladinosylazithromycin (DAZT) with the solvent front. The other ion-pairing agents used, namely triethanolamine and triethylamine, yielded poorer peak parameters compared to tetrabutylammonium hydroxide. To improve the resolution and peak symmetry factor of DAZT, the effect of the buffer concentration was investigated in the range of 10%–25% at 5% intervals. A buffer concentration of 15% was found to be optimal. In order to reduce the run time with baseline resolution of all components, the concentrations of acetonitrile and temperature were optimized to 25% *v*/*v* and 43 °C, respectively. Under these conditions, the peaks of all separated components had peak purities of unity.

**Figure 3:**
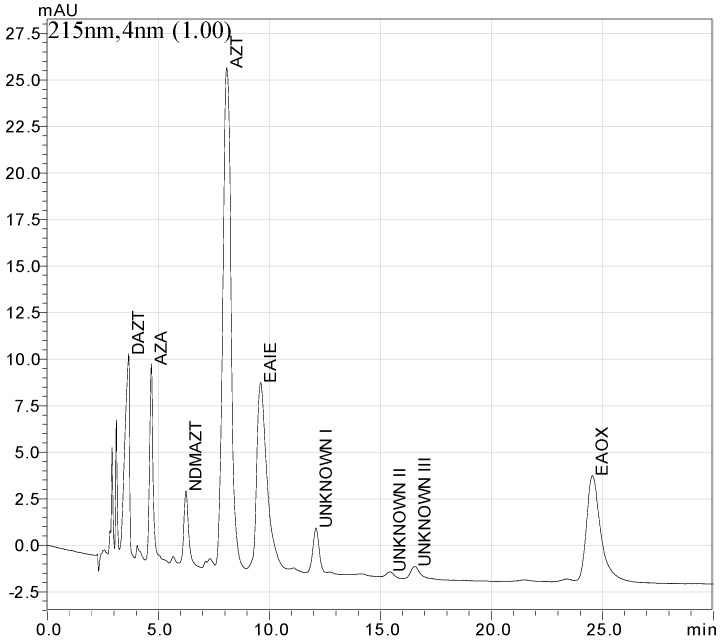
Typical chromatogram of the working mixture obtained under optimized chromatographic conditions.

The optimum chromatographic conditions for the separation of all components were established as a mobile phase comprised of acetonitrile-0.1 M KH_2_PO_4_ pH 6.5–0.1 M tetrabutyl ammonium hydroxide pH 6.5-water (25:15:1:59 *v*/*v*/*v*/*v*). Figure 3 shows a typical chromatogram obtained under the optimum conditions.

### 3.2. Method Validation

#### 3.2.1. Linearity

The linear regression equation was *y* = 9948*x* − 40457 with a coefficient of determination (*r*^2^) of 0.997, where *y* = peak area and *x* = concentration of the solution. The coefficients of variation (CV) of the peak areas obtained ranged between 0.02%–0.12% while the standard errors of the slope and intercept were 0.04 and 0.21, respectively. The developed method therefore met the ICH guidelines for linearity over the concentration range investigated [20,22].

#### 3.2.2. Sensitivity

When calculated against a sample solution of 5.0 mg/mL, the (the limit of dection) LOD was 0.02% (20 µg) (*n* = 6) at a S/N ratio of 3 while the limit of quantitation (LOQ) was 0.078% (78 µg) (*n* = 6) at a S/N ratio of 10. The LOD and LOQ results obtained demonstrated that the developed method was adequately sensitive for the determination of AZT and its related substances at the 0.1% level [20,22].

#### 3.2.3. Accuracy

The results for the recovery of azithromycin from spiked samples are shown in Table 2. The ICH guidelines for the validation of analytical procedures specify a mean recovery of 100% ± 2.0% at each concentration over the range of 80%–120% of nominal concentration [20]. The results shown in Table 2 indicate that the developed method is accurate with a mean recovery of 100.7%.

#### 3.2.4. Robustness

**Figure 4:**
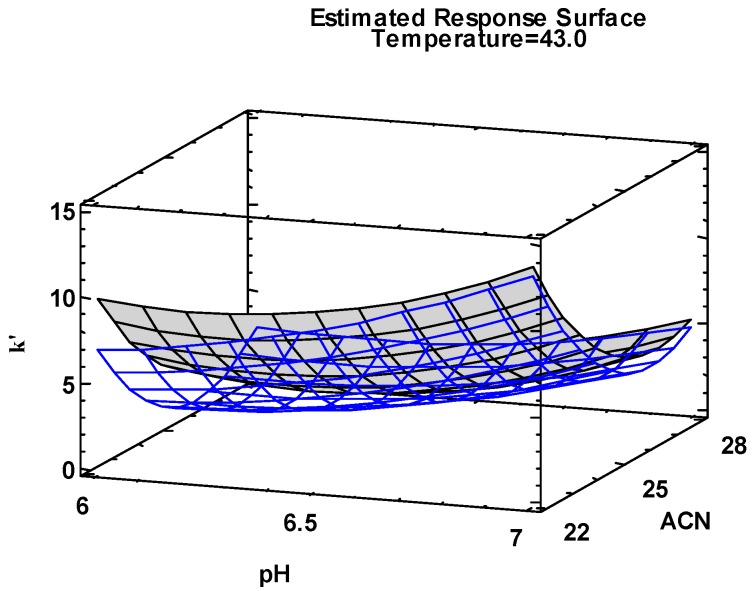
Overlaid response surfaces of AZT and EAIE. The blue and grey coloured surfaceslines represent the individual response surfaces of EAIE and AZT respectively.

The robustness ranges for pH were found to be pH 6–7, with 22%–28% *v*/*v* acetonitrile and 41–45 °C for the column temperature. Pareto analysis revealed that the acetonitrile concentration and buffer pH had the biggest impact on the separation of AZT and EAIE (*p* = 0.05). The response surface plot (Figure 4) showed the non-intersection of the response surface plots at all nominal values investigated, indicating that there was no co-elution of this critical peak. 

#### 3.2.5. Precision

The CV of the peak areas obtained from repeatability experiments was 0.97% while that for intermediate precision was in the range of 0.06%–0.11%. In all the determinations the CV was <1.5%, and thus the proposed method was found to be precise [20]. 

#### 3.2.6. Specificity

The resolution between azithromycin and all other components was >1.5, and thus the proposed method demonstrated sufficient specificity for analysis of azithromycin in accordance with ICH guidelines [20].

### 3.3. Forced Degradation of Azithromycin in Acidic and Oxidative Environments

The data generated from the oxidative degradation of azithromycin fitted into the second-order rate equation with the linear regression equation *y* = 0.0106*x* + 0.1377 (*r*^2^ = 0.994), a half life of 13 min and a rate constant (k) of 1.06 × 10^−2^ area units^−1^·min^−1^. This oxidative study may be indicative of the possible nature of degradation of AZT in the presence of oxidants and exposure to air during storage.

Acid degradation data showed that AZT degrades rapidly by about 90% within 10 min in 0.1 M H_3_PO_4_ and 0.05 M H_3_PO_4_ solutions maintained at 37 °C. However, at 0.025 M H_3_PO_4_ at 37 °C, the kinetics of AZT decomposition were derivable. The data generated fitted into the first-order rate equation with the linear regression equation *y* = −0.0002*x* + 13.651 (*r*^2^ = 0.9907), a half life of 57.8 h (3465 min) and a rate constant (k) of 2.0 × 10^−4^ min^−1^. In all instances, the degradation products in acid were well separated from the AZT peak, thus providing further evidence for the stability-indicating ability of the method. The degradation of AZT under acidic conditions is likely to affect oral bioavailability and efficacy of the drug due to gastric acid. For instance, with a mean gastric residence time for the AZT base as low as 10 min in gastric fluid of pH 1.6, 93% of the administered dose may be lost due to degradation. The degradation of AZT in gastric fluid may differ since there is a significant concentration gradient of the drug from gastric tissue to gastric juice [23].

### 3.4. Assay of Azithromycin Tablets and Suspensions

The developed and validated liquid chromatographic method was applied for the assay of azithromycin bulk samples and the oral dosage formulations of tablets and suspensions. The product excipients did not interfere with the determination of AZT in all the formulations analyzed. The peak purity index of AZT was confirmed to be unity by photo diode array analysis.

The United States Pharmacopeia (2015) limits for assay (90.0%–110.0% label claim) of azithromycin were adopted for quality assessment of the samples [13]. The assay test failure rates were 23% (*n* = 13) for tablet brands, 60% (*n* = 5) for premixed suspensions and 25% (*n* = 4) for dry powders (Table 3). There was a relatively higher assay test failure rate for premixed suspensions compared to dry powders for reconstitution. These findings underscore the need for generic manufacturers to carry out comprehensive formulation studies to justify any formulation aspects that significantly differ from those of the innovator products. The Drug Regulatory Authorities ought to evaluate supporting data for products that deviate from the innovator formulation to ensure that generics registered are pharmaceutically equivalent and bioequivalent to the innovator brand. Where significant changes in formulation occur, the generic manufacturer should carry out full clinical studies to support the rationale for such deviation [24].

The assay test failure rates observed underscore the need for continuous market surveillance on products available in the Kenyan market to ensure the circulation of good quality medicines. This will help curb the emergence of macrolide-resistant strains, thus preserving the antibiotic arsenal to treat susceptible microorganisms. Batch-to-batch variation of AZT content was not observed where different batches of the same product were analyzed (Table 3). 

## 4. Conclusions 

A selective, simple, specific, robust and isocratic liquid chromatographic method for the analysis of azithromycin in bulk samples and oral dosage forms was developed. The method met ICH guidelines for validation parameters. With a run time of 25 min, the method allows a relatively high sample throughput. Therefore, the method may be employed in the routine laboratory analysis of samples and stability studies. The presence of products that do not meet assay specifications in the market is a matter of public health concern and it underscores the need for sustained post-market surveillance by the Drug Regulatory Authority (DRA). This study did not encounter any counterfeits of AZT on the market.

## Figures and Tables

**Table 1:** Nominal values corresponding to low, central and high levels.

Factor	Low Level (−1)	Central Level (0)	High Level (+1)
pH	6.0	6.5	7.0
Acetonitrile (% vol.)	22	25	28
Temperature (°C)	41	43	45

**Table 2:** Recovery of azithromycin from spiked samples. CV; coefficient of variation.

Target Concentration (%)	Azithromycin				Mean Recovery (%)
	Amount Added (mg/mL)	Amount Recovered (mg/mL)	Recovery (Absolute %)	Recovery (CV %)	
80	4.98	4.975	99.9	0.16	100.7
100	5.08	5.171	101.8	0.20	
120	5.13	5.152	100.4	0.50	

**Table 3:** Assay results for selected commercial samples of azithromycin. The figures in parentheses represent the coefficient of variation; P, Powder for reconstitution; M, Premixed; a and b represent different batches of each product.

Product Code	Formulation	AZT Content (%)	Remarks
I	(a) Tablet	93.6 (1.6)	Complied
	(b) Tablet	94.8 (1.0)	Complied
II	(a) Tablet	110.3 (0.2)	Complied
	(b) Tablet	109.1 (0.1)	Complied
III	(a) Tablet	98.5 (0.5)	Complied
	(b) Tablet	99.2 (0.3)	Complied
IV	(a) Tablet	97.2 (0.2)	Complied
	(b) Tablet	97.6 (0.2)	Complied
V	Tablet	102.0 (1.5)	Complied
VI	(a) Tablet	109.9 (0.3)	Complied
	(b) Tablet	107.3 (0.3)	Complied
VII	(a) Tablet	105.1 (1.6)	Complied
	(b) Tablet	104.3 (1.3)	Complied
VIII	(a) Suspension (M)	110.0 (0.6)	Complied
	(b) Suspension (M)	109.3 (0.5)	Complied
IX	(a) Suspension (M)	96.4 (0.7)	Complied
	(b) Suspension (M)	96.1 (0.2)	Complied
X	Suspension (M)	69.3 (1.7)	Did not comply
XI	Suspension (M)	89.3 (0.4)	Did not comply
XII	Tablet	81.4 (0.5)	Did not comply
XIII	Tablet	99.8 (0.3)	Complied
XIV	Tablet	83.3 (0.2)	Did not comply
XV	Tablet	87.0 (1.1)	Did not comply
XVI	Tablet	103.3 (1.3)	Complied
XVII	Suspension (P)	88.6 (0.2)	Did not comply
XVIII	Suspension (P)	105.5 (0.4)	Complied
XIX	Suspension (M)	87.8 (1.3)	Did not comply
XX	Suspension (P)	98.7 (0.7)	Complied
Bulk sample	Powder	94.7 (0.1)	Complied
Zithromax^®^	Suspension (P)	99.6 (0.7)	Complied
Zithromax^®^	Tablet	96.4 (1.5)	Complied
